# Microvesicles-delivering Smad7 have advantages over microvesicles in suppressing fibroblast differentiation in a model of Peyronie’s disease

**DOI:** 10.1186/s12896-024-00866-1

**Published:** 2024-06-07

**Authors:** Wenting Wang, Fengchun Wan, Tianxi Yu, Shuang Wu, Xin Cui, Chongjun Xiang, Monong Li, Qingzuo Liu, Chunhua Lin

**Affiliations:** 1 Central Laboratory, The Affiliated Yantai Yuhuangding Hospital of Qingdao University, Yantai, 264000 China; 2 Organ Transplant Center, The Affiliated Yantai Yuhuangding Hospital of Qingdao University, Yantai, 264000 China; 3 School of Clinical Medicine, Weifang Medical University, Weifang, 261042 China; 4 Department of Urology, The Affiliated Yantai Yuhuangding Hospital of Qingdao University, Yantai, 264000 China; 5 The 2nd Medical College of Binzhou Medical University, Yantai, 264003 China; 6 Department of Urology, Qingdao Municipal Hospital, Qingdao, 266011 China

**Keywords:** Microvesicles, Fibroblast differentiation, M1 macrophages, Peyronie’s disease

## Abstract

**Background:**

This study compared the differences of microvesicles (MVs) and microvesicles-delivering Smad7 (Smad7-MVs) on macrophage M1 polarization and fibroblast differentiation in a model of Peyronie’s disease (PD).

**Methods:**

Overexpression of Smad7 in rat BMSCs was obtained by pCMV5-Smad7 transfection. MVs were collected from rat BMSCs using ultracentrifugation. In cells, 100 µg/mL of MVs or Smad7-MVs were used to treat the 100 ng/mL of lipopolysaccharide (LPS)-induced RAW264.7 cells or 10 ng/mL of recombinant transforming growth factor-β1 (TGF-β1)-induced fibroblasts. The pro-inflammatory cytokines and markers of M1 macrophages were measured in RAW264.7 cells, and the migration and markers of fibroblast differentiation were measured in fibroblasts. In rats, 50 µg of MVs or Smad7-MVs were used to treat the TGF-β1-induced animals. The pathology of tunica albuginea (TA), the markers of M1 macrophages and fibroblast differentiation in the TA were measured.

**Results:**

The MVs or Smad7-MVs treatment suppressed the LPS-induced macrophage M1 polarization and TGF-β1-induced fibroblast differentiation. Moreover, the Smad7-MVs treatment decreased the fibroblast differentiation compared with the MVs treatment. In the TGF-β1-induced TA of rats, MVs or Smad7-MVs treatment ameliorated the TA fibrosis by suppressing the macrophage M1 polarization and fibroblast differentiation. There was no significance on the M1-polarized macrophages between the MVs treatment and the Smad7-MVs treatment. Meanwhile, the Smad7-MVs treatment had an edge in terms of suppressing the fibroblast differentiation in the TGF-β1-induced PD model compared with the MVs treatment.

**Conclusions:**

This study demonstrated that Smad7-MVs treatment had advantages over MVs treatment in suppressing of fibroblast differentiation in a model of PD.

**Supplementary Information:**

The online version contains supplementary material available at 10.1186/s12896-024-00866-1.

## Introduction

Peyronie’s disease (PD) is characterized by multiple fibrous nodules in either tunica albuginea (TA) or corpora cavernosa, occurring in men aged 40–60 years [1]. As the plaques grow, they will lead to the pain and the deformity while erection, seriously affecting personal physical and mental health, such as erectile function, self-esteem, and the psychological disorders [1, 2]. At present, there is no strong data to support any oral agents as monotherapy for PD, but intralesional injection of agents improves, including natural products, stem cell therapy, and platelet-derived preparations, but does not get rid of [2–4]. It is highly desirable to find more effective agents by intralesional injection to relieve symptoms of PD.

In the progression of PD, transforming growth factor-β1 (TGF-β1) shows accumulation in TA, and injection of TGF-β1-specfifc inhibitor can improve the fibrotic progression of TA [3, 5–7]. Researchers have studied the reasons of abnormal TGF-β1 expression, and consider that the extravasation of fibrin from the microvasculature of TA promotes the TGF-β1 expression [8, 9]. High levels of TGF-β1 contribute to fibroblasts differentiate into myofibroblasts in the TA though increasing phosphor(p)-Smad2/Smad3 protein and decreasing Smad7 protein [10–12]. Smads are the main components of the TGF-β1 pathway [10, 13]. Fibroblasts can secrete collagen fibers, elastic fibers, reticular fibers and organic matrix, etc., which contribute to elasticity of TA [14]. Under pathological conditions, fibroblasts can transform into myofibroblasts to secrete a large amount of collagen and fibronectin [12, 14, 15]. Therefore, suppressing the TGF-β1-induced fibroblast differentiation is important for the PD treatment.

In the active phase of PD, a large number of neutrophils infiltrate the penis, and release many inflammatory factors, that forms an inflammatory microenvironment [16, 17]. Macrophages exist two distinct subsets: M1 macrophages and M2 macrophages, which are regulated by the surrounding microenvironment [18]. Pro-inflammatory cytokines, such as interleukin (IL)-1β, IL-6, CD86, inducible nitric oxide synthase (iNOS) and tumor necrosis factor (TNF)-α, are produced by M1 macrophages. However, anti-inflammatory cytokines such as IL-10, CD206, arginase1 (Arg1) and TGF-β, are produced by M2 macrophages. The balance of M1/M2 macrophages has significant impact on organs [18, 19]. The dysfunction of macrophages can lead to abnormal repair, such as uncontrolled production of inflammatory mediators and growth factors. These uncontrolled factors lead to communication failure among cells, promoting a state of sustained damage [20]. Therefore, suppressing the macrophage M1 polarization is also important for the PD treatment.

In our previous study [1], we have found that intralesional injection of rat-derived bone marrow mesenchymal stem cells (BMSCs) can improve fibrosis of penile TA in the PD rat, and the mechanism is associated with Smad7 expression in the fibroblasts. However, a large number of stem cells with potential carcinogenic risks prevent their clinical applications [21]. Microvesicles (MVs, 100–1000 nm) are small lipid membrane vesicles derived from cells, which play key roles in cell adhesion, motility, membrane fusion, signaling, and protein trafficking [22]. Comparison to the BMSCs, BMSCs-derived MVs exhibit significant advantages with less toxicity, minimal immune rejection and circulation stability [21, 23]. Based on our previous finding, we selected the BMSCs-derived MVs to study the therapeutic effects of MVs on the model of PD.

In this study, the MVs or MVs-delivering Smad7 (Smad7-MVs) were used to treat the LPS-induced RAW264.7 and TGF-β1-induced fibroblasts to observe their effects on the macrophage M1 polarization and fibroblast differentiation. Moreover, the MVs or Smad7-MVs were used to treat the TGF-β1-induced rats to observe their effects on the TA fibrosis.

## Materials and methods

### Cell culture

Rat BMSCs (#RASMX-01001, OriCell Sprague-Dawley, Cyagen) were cultured in its complete medium (#RASMX90011, Cyagen). Short tandem repeat (STR)-identified RAW264.7 cells (#CL-0190, Procell) were cultured in Dulbecco’s modified eagle medium (DMEM). Fibroblasts derived from rat penile TA (#CP-R316, Procell) were cultured in its complete culture medium (#CM-R316, Procell). All cells were cultured at 37℃ and 5% CO_2_. At about 80% confluence (2 × 10^4^ per well), cells were used for experiments.

### BMSCs transfection

According to previous methods [10], the serum starvation-induced BMSCs were transfected with 1 µg PEI25k/pCMV5-Smad7 vector or empty PEI25k/pCMV5 vector (Addgene, Cambridge, MA, USA) for 24 h. The transfected BMSCs were cultured for 48 h.

### Isolation of BMSCs-derived MVs by ultracentrifugation

According to reported protocols [24, 25], the BMSCs-derived MVs were isolated from cell culture supernatants by ultracentrifugation. In brief, the supernatants were centrifugated at 2,500 *× g* for 5 min, and then collected at 10,000 *× g* for 30 min at 4℃. Phosphate buffer saline (PBS) was used to suspend the particles, and the particles were centrifuged (100,000 *× g*, 120 min) twice at 4℃. The MVs were suspended in PBS and stored at -20℃. The structure of MVs was observed by a transmission electron microscopy (TEM, FEI Tecnai Spirit TEM T12, USA) and the diameter of MVs was analyzed by a qNano particle analyzer (#ZEN3690, Malvern instruments limited, UK). The specific MVs markers, CD9 and CD63, were measured by western blotting. The delivery of Smad7 in MVs was assessed by western blotting.

### Biocompatibility of Smad7-MVs in cells

The biocompatibility of Smad7-MVs was observed using fluorescence labeling [26]. In brief, the MVs were labeled using 1 µM Alexa Fluor 647 (red, #P0180, Beyotime, China) for 30 min. After washing with PBS, the MVs were collected using ultracentrifugation (120, 000 × g, 60 min, 4℃). Cells were labeled using 8 µM PKH67 (green, #Mini67, Sigma) for 15 min, then respectively cultured with 1 µg/mL MVs for 2 h, 12 h and 24 h. The nuclei of cells were stained with 4′,6-diamidino-2-phenylindole (DAPI) for 5 min. After washing with PBS, the cells were collected (800 × g, 10 min) and observed under a confocal microscope (#LSM800, Zeiss, Germany).

### Cell model and treatments

RAW264.7 cells were stimulated with 100 ng/mL lipopolysaccharides (LPS, #HY-D1056, MedChemExpress, China) for 24 h, and cocultured with 100 µg/mL of MVs or Smad7-MVs for 24 h at 37℃ and 5% CO_2_. Cells were treated with equal volume of PBS as controls. The levels of IL-6, IL-1β and TNF-α in cell supernatants were measured using enzyme-linked immunosorbent assay (ELISA), and markers of M1 macrophages, CD86 and iNOS, were measured using flow cytometry and immunofluorescence.

Fibroblasts were stimulated with 10 ng/mL TGF-β1 (#HY-P7118, MedChemExpress, China) for 24 h, and cocultured with 100 µg/mL of MVs or Smad7-MVs for 24 h. Cells were treated with equal volume of PBS as controls. Migration of cells was measured using Transwell. The markers of fibroblast differentiation, α-smooth muscle actin (SMA) and collagen III, were measured using immunofluorescence and western blotting.

### Animals

Twenty-four male Sprague-Dawley rats (11–12 weeks old, 300–350 g) were used in this study. All animals were housed under specific pathogen free conditions with 20–22 °C, 45–55% humidity and a12 h/12 h light/dark cycle. All rats were freely available food and water.

### Animal model and treatments

According to reported methods [27], the rats were anaesthetized with propofol (26 mg/kg) intraperitoneal injection and injected with 50 µL TGF-β1 (0.5 µg) in the right midshaft dorsomedial TA using a microlitre syringe. Animals were divided into four groups: sham group, PD group, PD + MVs group and PD + Smad7-MV group. In the sham group, rats were injected with equal volume of PBS. In the PD + MVs group or PD + Smad7-MV group, rats were injected with 50 µL TGF-β1, then injected with 50 µL (50 µg) of MVs or Smad7-MVs next day. The TGF-β1 and MVs were dissolved in PBS. After 4 weeks, all rats were sacrificed using pentobarbital sodium (150 mg/kg) intraperitoneal injection to collect penises. The penises were fixed with 4% paraformaldehyde.

### Western blotting

According to reported procedures [28], levels of Smad7, α-SMA and collagen III proteins were measured using western blotting. The primary antibodies targeting to Smad7 (1:1000, #AF5147), α-SMA (1:1000, #AF1032), collagen III (1:1000, #AF5457) and β-actin (1:10000, #AF7018) were purchased from Affinity Biosciences (Jiangsu, China). The secondary antibody anti-rabbit IgG (H + L) (1:500, BM3894) was purchased from Boster (Wuhan, China).

### ELISA

Cell supernatants were collected from RAW246.7 cells by centrifugation (800 *× g*, 15 min). The levels of IL-6 (#PI326), IL-1β (#PI301) and TNF-α (#PT512) in cell supernatants were measured by ELISA kits. The kits were obtained from the Beyotime (Shanghai, China).

### Flow cytometry

M1 polarization of RAW264.7 was analyzed using flow cytometry. Cells (2 × 10^5^) were respectively incubated with 5 µL FITC anti-rat CD86 antibody (#200,305, BioLegend, USA) and 5 µL APC anti-iNOS antibody (#696,807, BioLegend, USA) for 30 min without light at 37 °C. After dilution with PBS (550 µL), the results were obtained within 60 min through a flow cytometry (CytoFlex, Beckman).

### Immunofluorescence

According to reported procedures [29], cells (10^4^/well) were fixed with 4% paraformaldehyde for 10 min and blocked with BS-T containing 1% BSA (TBS) for 60 min at 25 °C. RAW264.7 were respectively incubated with iNOS antibody (1:200, #696,807, BioLegend) and CD86 antibody (1:200, #200,305, BioLegend) overnight at 4℃. Fibroblasts were respectively incubated with α-SMA antibody (1:200, #AF1032, Affinity Biosciences, China) and collagen III antibody (1:200, #AF5457, Affinity Biosciences) overnight at 4℃. After washing, the cells were respectively incubated with FITC-conjugated antibody (1:2000, #ab6717, Abcam) or Cy3-conjugated antibody (1:2000, #ab6939, Abcam) for 120 min. The nuclei of cells were stained with DAPI for 60s.

### Transwell

According to reported methods [30], migration of fibroblasts was measured using Transwell assay.

### Pathology observation of TA

Pathology of TA was analyzed by haematoxylin-eosin (H&E) staining (#C0105S, Beyotime, China) and Masson staining (#G1340, Solarbio, China).

### Immunohistochemistry

According to reported procedures [29], the sections (4 μm) were respectively incubated with primary antibodies overnight at 4℃. After washing, the sections were incubated with the secondary antibody (1:500, #S0001) for 120 min at 25 °C. The primary antibodies included Smad7 (1:200, #AF5147), p-Smad3 (1:200, #AF8315), iNOS (1:200, #AF0199), IL-1β (1:200, #AF5103), α-SMA (1:200, #AF1032) and collagen III (1:200, #AF5457). The antibodies were obtained from the Affinity Biosciences (Jiangsu, China).

### Statistics

IBM SPSS software (v. 20.0, National Institutes of Health, US) was used to analyze the data, and the results showed as mean ± standard deviation. One-way analysis of variance following the Scheffe’s posthoc test was use to assess significance among groups. *P* < 0.05 represents statistical significance.

## Results

### MVs or Smad7-MVs treatment suppressed the macrophage M1 polarization in the LPS-induced RAW264.7

The characterizations of MVs or Smad7-MVs were confirmed in Supplemental Fig. 1. The MVs showed cup-shaped or spherical morphology (Supplemental Fig. 1A), and the diameter of MVs was ranging from 20 to 250 nm (Supplemental Fig. 1B). The specific MVs markers, CD9 and CD63, were observed in the MVs (Supplemental Fig. 1C). Moreover, higher levels of Smad7 protein in the Smad7-MVs were observed compared with the MVs (Supplemental Fig. 2). A good biocompatibility of Smad7-MVs was found in RAW264.7 (Supplemental Fig. 3) and fibroblasts (Supplemental Fig. 4). As time went by, the Smad7-MVs entered the cells.

**Fig. 1:**
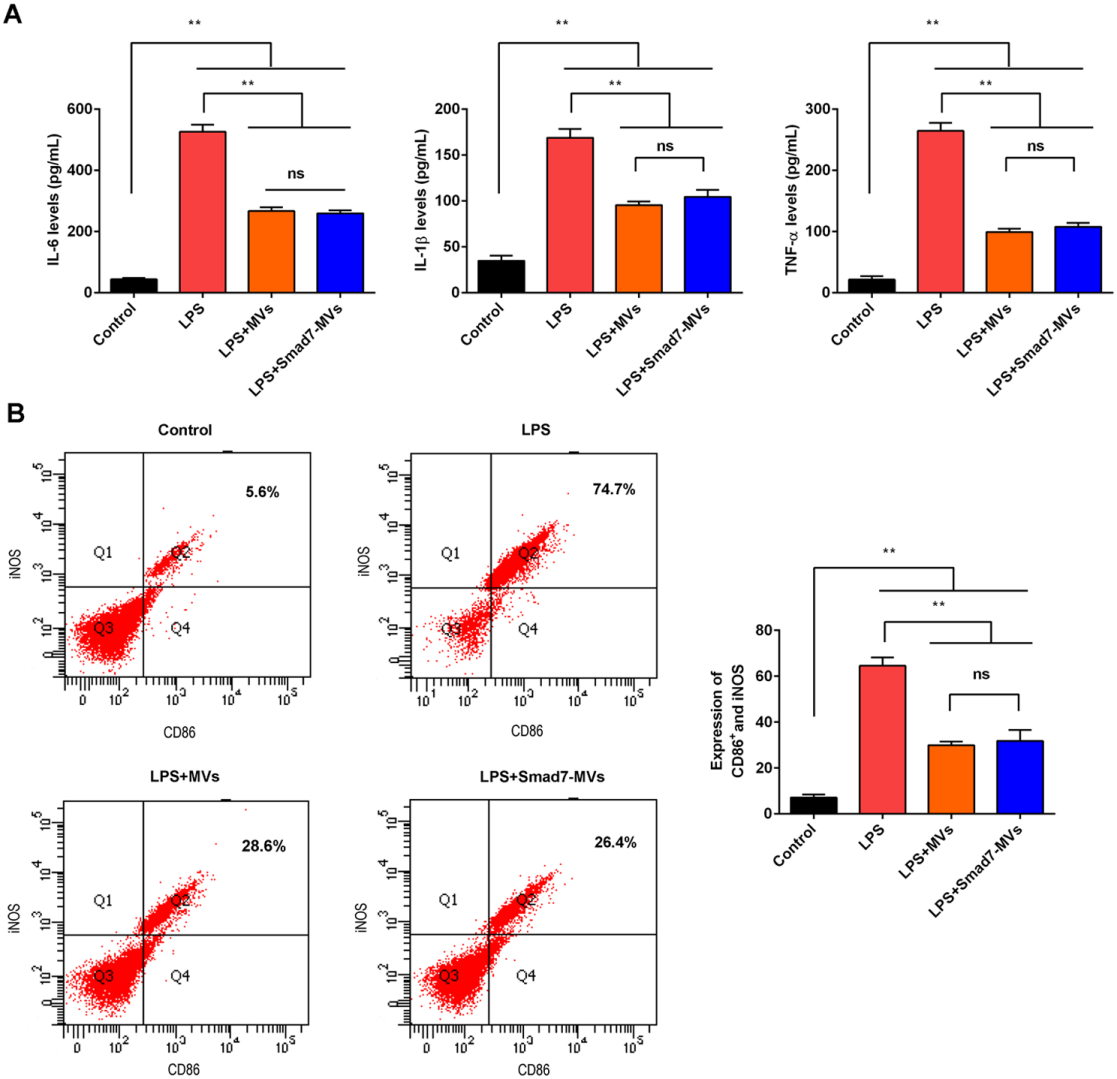
Effects of MVs or Smad7-MVs treatment on macrophage M1 polarization in the LPS-stimulated RAW264.7. RAW264.7 cells were treated with 100 ng/mL LPS for 24 h, and cocultured with 100 µg/mL MVs or Smad7-MVs for 24 h. **(A)** Levels of IL-6, IL-1β and TNF-α in cell supernatants. **(B)** Numbers of cells expressing CD86 and iNOS were analyzed using flow cytometry. ^**^p < 0.01. ns: no significance

The effects of MVs or Smad7-MVs treatment on LPS-treated RAW264.7 were observed. As shown in Fig. 1, the levels of M1 macrophage markers, IL-6, IL-1β and TNF-α (Fig. 1A), were significantly increased after the 100 ng/mL LPS stimulation compared with the control cells (*p* < 0.01). The 100 µg/mL of MVs or Smad7-MVs treatment significantly decreased the levels of IL-6, IL-1β and TNF-α in the LPS-induced RAW264.7 (*p* < 0.01), and no significance was found between the MVs treatment and Smad7-MVs treatment. Additionally, the M1 macrophage markers, CD86 and iNOS, were measured using flow cytometry (Fig. 1B). It showed that the numbers of cells expressing CD86 and iNOS were significantly increased after LPS treatment compared with the control cells (*p* < 0.01). The MVs or Smad7-MVs treatment significantly decreased the numbers of cells expressing CD86 and iNOS in the LPS-induced RAW264.7(*p* < 0.01), and no significance was found between the MVs treatment and Smad7-MVs treatment.

**Fig. 2:**
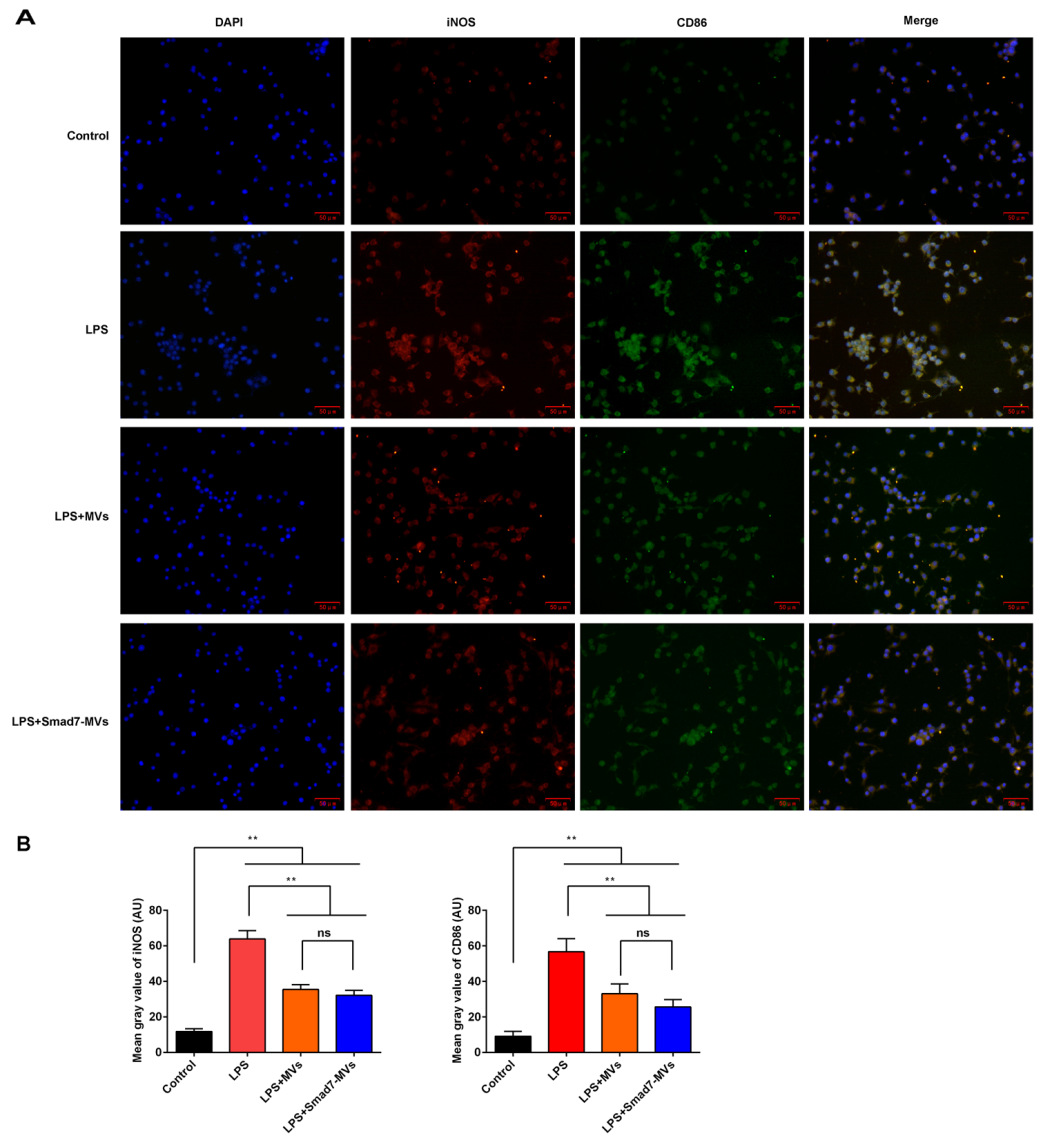
Effects of MVs or Smad7-MVs treatment on expressions of iNOS and CD86 in the LPS-induced RAW264.7. **(A)** Expressions of iNOS and CD86 were observed using immunocytochemistry in the RAW264.7. Scale = 50 μm. **(B)** About 700 cells have been analysed to represent the mean gray values. Mean gray values of iNOS and CD86 were analyzed using ImageJ software. ^**^p < 0.01. ns: no significance

The expressions of iNOS and CD86 were also observed in the cells using immunofluorescence (Fig. 2A). Compared with the control cells, the LPS administration significantly increased the expression of iNOS and CD86 (*p* < 0.01, Fig. 2B). MVs or Smad7-MVs treatment significantly suppressed the expression of CD86 and iNOS in the LPS-induced cells (*p* < 0.01). No significance was found between the MVs treatment and the Smad7-MVs treatment.

### Smad7-MVs had advantages over MVs in suppressing fibroblast differentiation in the TGF-β1-induced fibroblasts

**Fig. 3:**
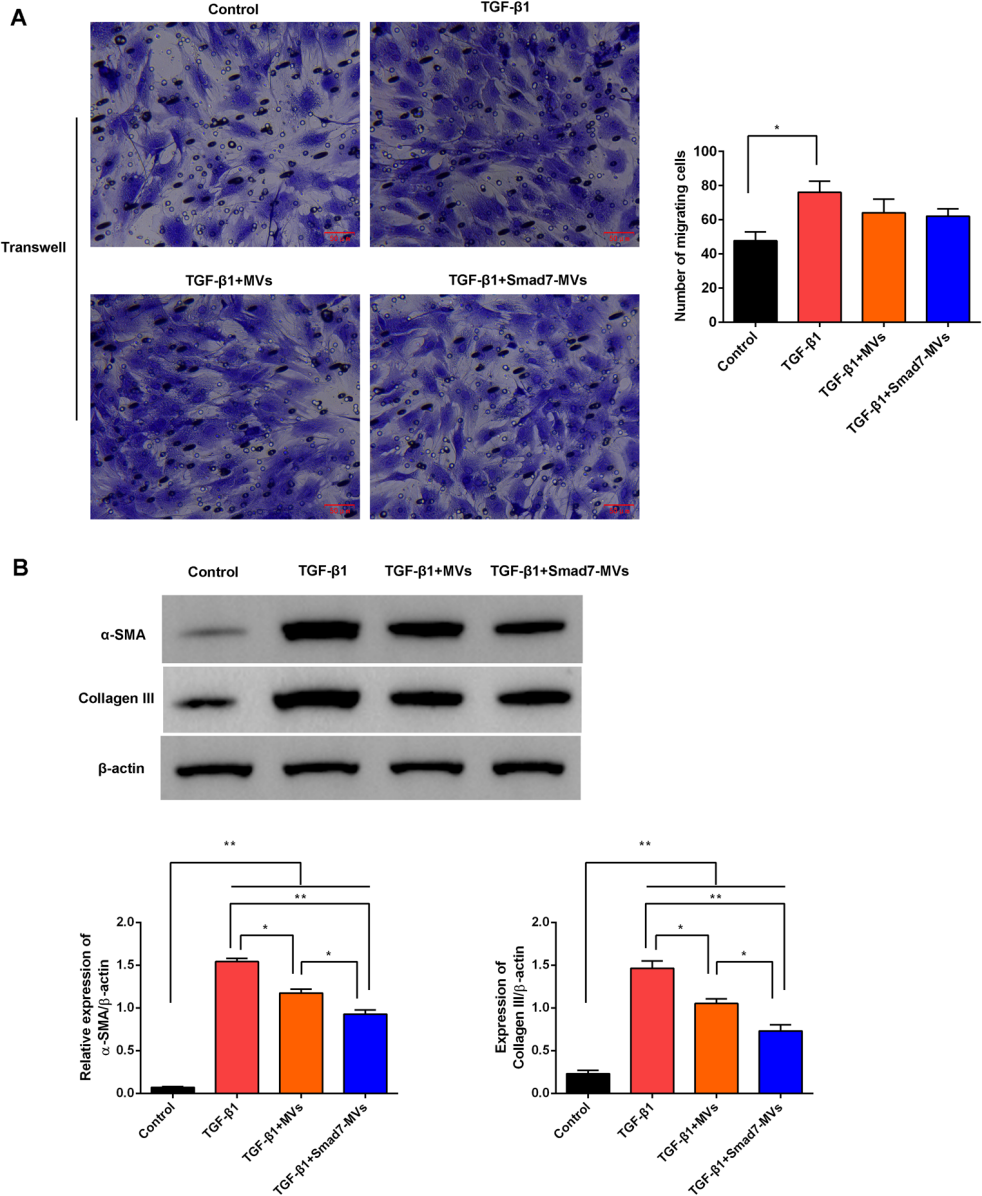
Effects of MVs or Smad7-MVs treatment on the fibroblast differentiation in the TGF-β1-stimulated fibroblasts. Fibroblasts were treated with 10 ng/mL TGF-β1 for 24 h, and cocultured with 100 µg/mL MVs or Smad7-MVs for 24 h. **(A)** Migration of fibroblasts was observed using Transwell. **(B)** Expressions of α-SMA and collagen III protein were analyzed using Western blotting. ^*^p < 0.05, ^**^p < 0.01

The effects of MVs or Smad7-MVs treatment on TGF-β1-induced fibroblasts were observed. As shown in Fig. 3A, the migration of fibroblasts increased after the 10 ng/mL of TGF-β1 simulation compared with the control cells (*p* < 0.05). However, the cell migration was not significantly decreased in the TGF-β1-stimulated cells after the 100 µg/mL of MVs or Smad7- MVs treatment. The makers of fibroblast differentiation α-SMA and collagen III were measured, and the results showed that the levels of α-SMA and collagen III protein (Fig. 3B) were increased after the TGF-β1 stimulation compared with the control cells (*p* < 0.01). MVs or Smad7-MVs treatment significantly decreased the levels of α-SMA and collagen III protein in the TGF-β1-induced fibroblasts (*p* < 0.05). Moreover, the Smad7-MVs treatment further decreased the levels of α-SMA and collagen III protein compared with the MVs treatment (*p* < 0.05).

**Fig. 4:**
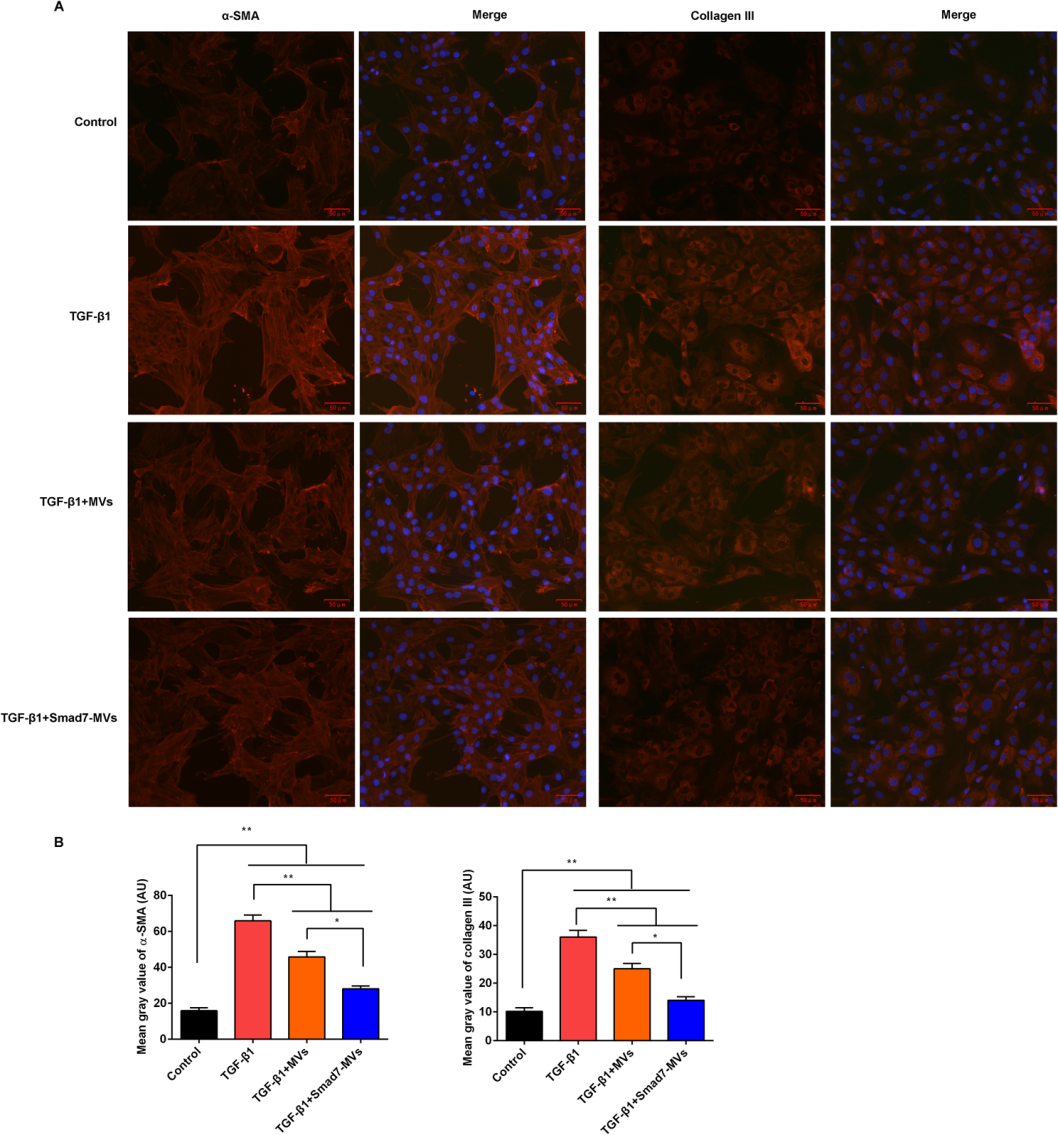
Effects of MVs or Smad7-MVs treatment on expressions of α-SMA and collagen III in TGF-β1-induced fibroblasts. **(A)** Expressions of α-SMA and collagen III were analyzed using immunocytochemistry, scale = 50 μm. **(B)** About 700 cells have been analysed to represent the mean gray values. Mean gray value of α-SMA and collagen III was analyzed using ImageJ software. ^*^p < 0.05, ^**^p < 0.01

Additionally, the expressions of α-SMA and collagen III in the fibroblasts were measured using immunofluorescence (Fig. 4A). The mean gray values of α-SMA and collagen III (Fig. 4B) were increased after the TGF-β1 simulation compared with the control cells (*p* < 0.01). MVs or Smad7-MVs treatment significantly declined the mean gray values of α-SMA and collagen III in the TGF-β1-stimualted fibroblasts (*p* < 0.01). And, the Smad7-MVs treatment further decreased the mean gray values of α-SMA and collagen III compared with the MVs treatment (*p* < 0.05).

### Smad7-MVs had advantages over MVs in ameliorating the TA fibrosis in a rat model of PD

**Fig. 5:**
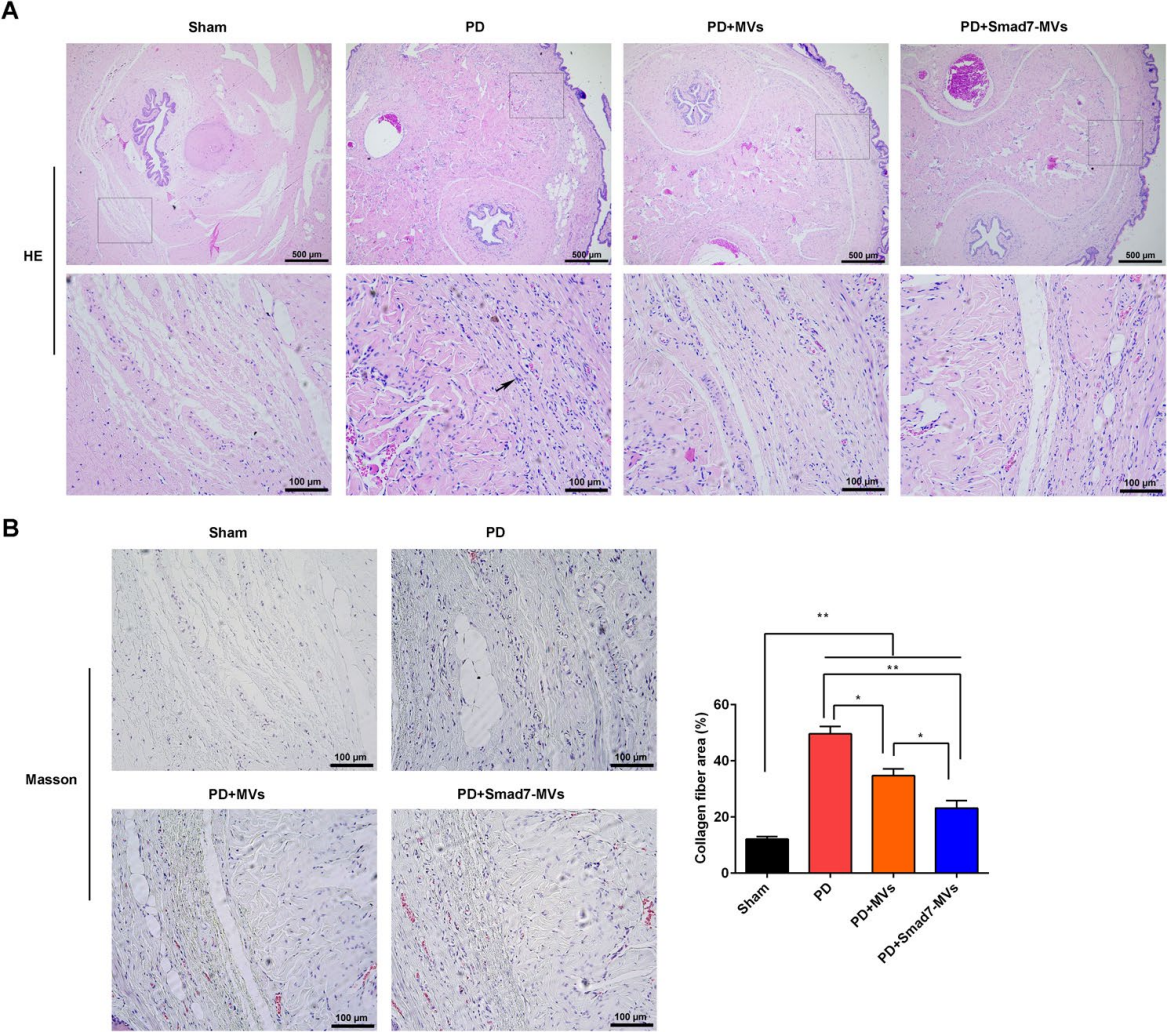
Effects of MVs or Smad7-MVs treatment on the changes of pathology in the TA of rats. 50 µL TGF-β1 (0.5 µg) were injected into the right midshaft dorsomedial TA. After injection one day, the TA was injected with 50 µL (50 µg) MVs or Smad7-MVs. The changes of pathology of TA were observed using H&E **(A)** and Masson **(B)**. Scale = 100 μm. Black arrow: inflammatory factor. ^*^p < 0.05, ^**^p < 0.01

A rat model of PD was induced by injection of TGF-β1, and then treated with MVs or Smad7-MVs. The pathological changes of TA were observed using H&E (Fig. 5A) and Masson (Fig. 5B). It showed that the TGF-β1 injection induced inflammation in the TA (black arrows), and MVs or Smad7-MVs treatment decreased the inflammation in the TA. In the meantime, the area of collagen fibers in the TA was increased after the TGF-β1 injection compared to the sham rats (*p* < 0.01). MVs or Smad7-MVs treatment decreased the area of collagen fibers in the TGF-β1-injected TA compared with the PD rats (*p* < 0.05). Furthermore, the Smad7-MVs injection declined the area of collagen fibers compared with the MVs injection (*p* < 0.05).

### Smad7-MVs treatment regulated Smad pathway in a rat model of PD

**Fig. 6:**
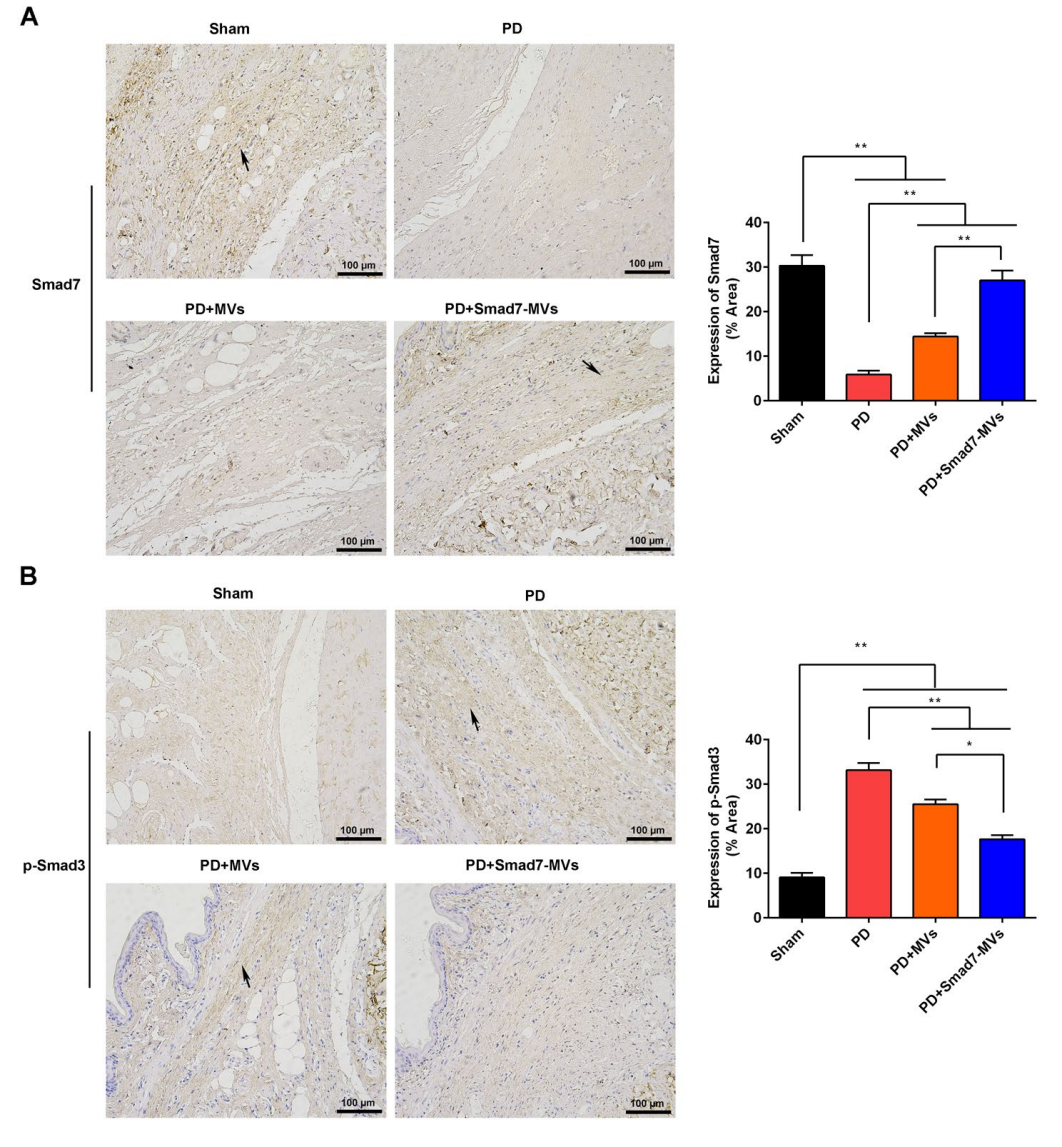
Effects of MVs or Smad7-MVs treatment on the Smad pathway in the TA of rats. Expressions of Smad7 **(A)** and p-Smad3 **(B)** in the TA were observed using immunohistochemistry. Scale = 100 μm. Black arrows: positive expressions. ^*^p < 0.05, ^**^p < 0.01

The expressions of Smad7 (Fig. 6A) and p-Smad3 (Fig. 6B) in the TA of rats were observed using immunohistochemistry. The results showed that the TGF-β1 injection decreased the Smad7 expression and increased the p-Smad3 expression in the TA of rats compared with the sham rats (*p* < 0.01). In the TGF-β1-injected TA, MVs or Smad7-MVs treatment increased the Smad7 expression and decreased the p-Smad3 expression compared with the PD rats (*p* < 0.01), and the effects of Smad7-MVs treatment were better than the MVs treatment (*p* < 0.05).

### MVs or Smad7-MVs treatment suppressed the macrophage M1 polarization in a rat model of PD

**Fig. 7:**
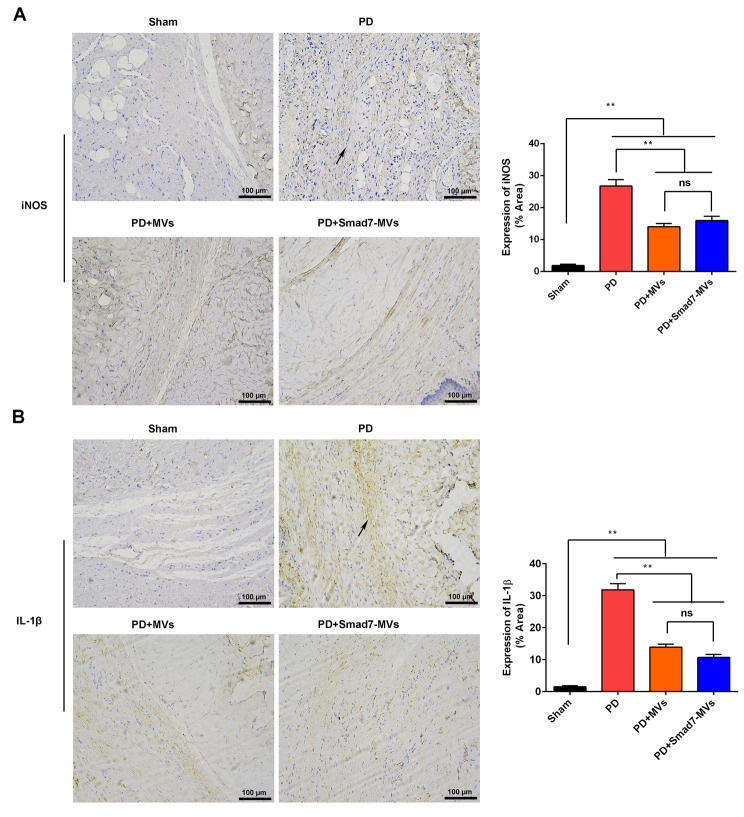
Effects of MVs or Smad7-MVs treatment on expressions of iNOS and IL-1β in the TA of rats. The expressions of iNOS **(A)** and IL-1β **(B)** in the TA were measured using immunohistochemistry. Scale = 100 μm. Black arrows: positive expressions. ^**^p < 0.01, ns: no significance

The markers of M1 macrophages, iNOS (Fig. 7A) and IL-1β (Fig. 7B), in the TA were measured using immunohistochemistry. It showed that the TGF-β1 injection increased the expressions of iNOS and IL-1β in the TA compared with the sham rats (*p* < 0.01). MVs or Smad7-MVs treatment decreased the expressions of iNOS and IL-1β in the TGF-β1-injected TA compared with the PD rats (*p* < 0.01). However, there was no significance between the MVs treatment and Smad7-MVstreatment.

### Smad7-MVs had advantages over MVs in suppressing fibroblast differentiation in a rat model of PD

**Fig. 8:**
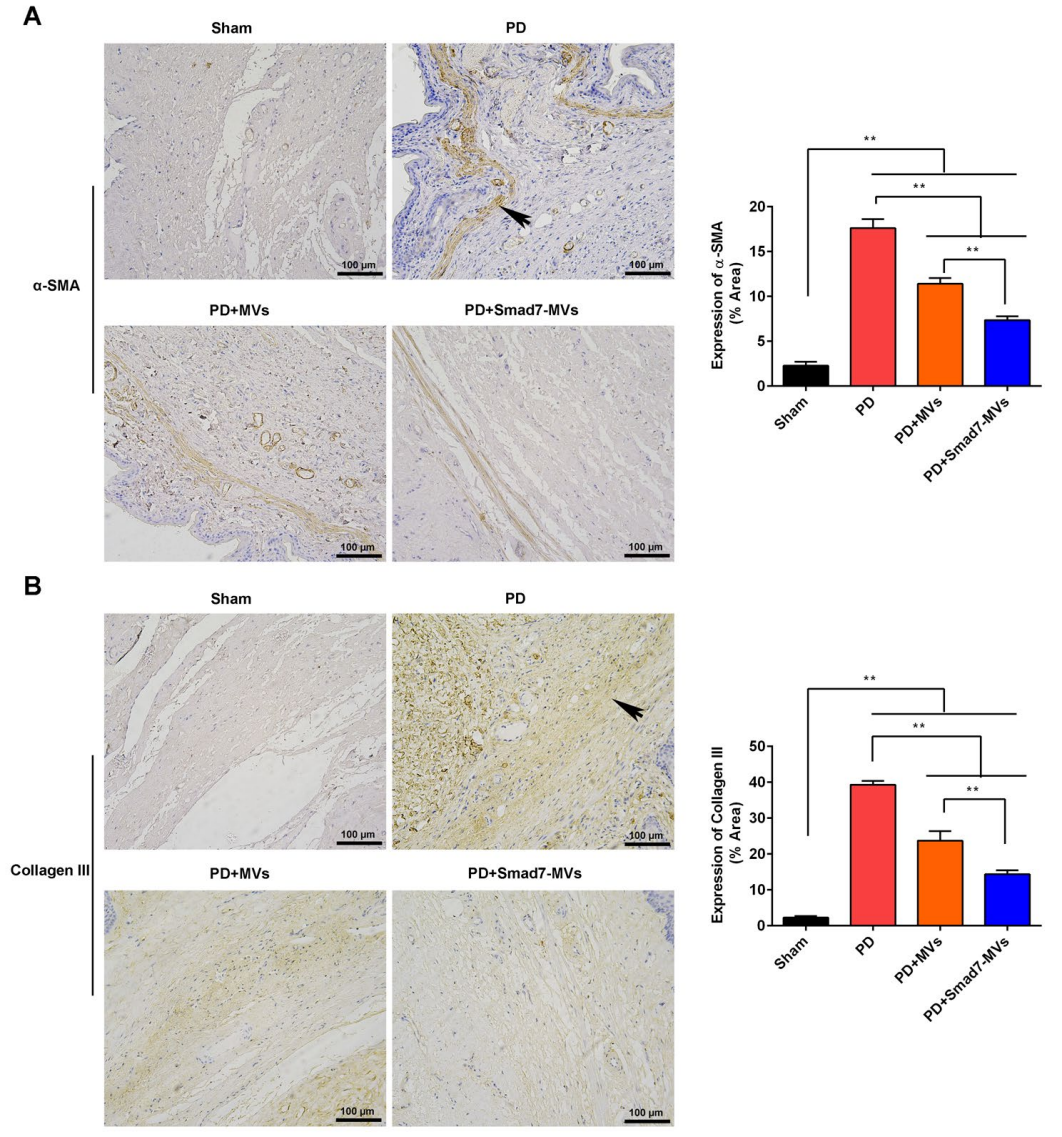
Effects of MVs or Smad7-MVs treatment on the expressions of α-SMA and collagen III in the TA of rats. The expressions of α-SMA **(A)** and collagen III **(B)** in the TA were measured using immunohistochemistry. Scale = 100 μm. Black arrows: positive expressions. ^**^p < 0.01

The markers of fibroblast differentiation, α-SMA (Fig. 8A) and collagen III (Fig. 8B), were measured in the TA using immunohistochemistry. By comparation with the sham rats, the TGF-β1 injection increased the expressions of α-SMA and collagen III in the TA (*p* < 0.01). MVs or Smad7-MVs treatment decreased the expressions of α-SMA and collagen III in the TGF-β1-injected TA compared with the PD rats (*p* < 0.01). Moreover, the Smad7-MVs treatment further decreased the expressions of α-SMA and collagen III in the TGF-β1-injected TA compared with the MVs treatment (*p* < 0.01).

## Discussion

MVs derived from stem cells have become substitutes for stem cell therapy due to their low immunogenicity, good tolerance and high delivery efficiency [31]. In this study, the isolated Smad7-MVs are nanometer-sized membrane vesicles (20–250 nm) with a good bioavailability in RAW264.7 and fibroblasts. Furthermore, the levels of Smad7 protein were increased in the Smad7-MVs compared with MVs. It has been reported that intratunical injection of exosomes derived from human urine-derived stem cells (USC-exo) can improve erectile function and ameliorate the TA fibrosis in the TGF-β1-induced PD rat model [27]. The mechanism of USC-exo may be associated with the inhibition of differentiation of fibroblasts into myofibroblasts [27]. Consistent with this finding, our study showed that MVs or Smad7-MVs treatment suppressed the fibroblast differentiation through regulating the TGF-β-mediated Smad pathway in the model of PD.

As a negative regulator of TGF-β signaling, Smad7 can suppress the phosphorylation and recruitment of Smad2/3 [32]. The TGF-β-mediated fibrotic responses are initiated by activation of Smad2/3 upon ligand binding, and further induce fibroblasts differentiation into myofibroblasts [10]. Fibroblast differentiation is characterized by the contractile stress fibers formation, which secrete a large amount of collagen and fibronectin, including α-SMA and collagen III [33, 34]. It has been found that overexpression of Smad7 reduce the expression of extracellular matrix proteins, such as fibronectin, collagen I and collagen IV, thus inhibiting the TGF-β1-mediated fibrotic response of fibroblasts derived from human PD plaques [10]. In our previous study [1], BMSCs treatment increased the Smad7 expression in the model of PD to suppress the TGF-β1-mediated fibroblast differentiation. Here, we found that the Smad7-MVs treatment had an edge in terms of suppressing the fibroblast differentiation in the TGF-β1-induced PD model compared with the MVs treatment.

Generally, the inflammatory response and extra-cellular matrix (ECM) deposition induced by various traumatic factors constitute the normal reparative process. In the progression of PD, the penile trauma provokes a delamination of the tunica albuginea with a consequent small hematoma, which successively progresses as inflammation and the subsequent accumulation of inflammatory cells [35]. Fibrin act indeed as a strong chemoattractant, promoting the inflow of inflammatory cells such as macrophages, neutrophils, and cytokines. In the inflammatory process of PD, the M1-polarized macrophages and proinflammatory cytokines are important triggering factors [4]. Natural products (such as polyphenols), stem cell therapy, and platelet-derived preparations have provided positive results, including improvement of penile function, reduce of inflammation and oxidative stress, and promotion of tissue repair in the model of PD [4]. Consistent with these findings, this study also showed that MVs or Smad7-MVs suppressed the M1-polarized macrophages in the LPS-induced RAW264.7 and the TGF-β1-stimulated rats. Importantly, there was no significance on the M1-polarized macrophages between the MVs treatment and the Smad7-MVs treatment. This finding suggests that the mechanism of MVs on suppression of M1-polarized macrophages is not directly associated with Smad pathway.

However, it is unclear there are other growth factors and transcription factors present in the MVs that may be impacting PD fibrosis. In addition, the impacts of Smad7 on other factors in the MVs are unclear. More studies are needed to be performed in the future.

## Conclusions

In this study, it showed that Smad7-MVs or MVs treatment suppressed M1-polarized macrophages and fibroblast differentiation in a model of PD. Moreover, the Smad7-MVs treatment has an edge in terms of suppressing the fibroblast differentiation in the TGF-β1-induced PD model compared with the MVs treatment.

### Electronic supplementary material

Below is the link to the electronic supplementary material.

Supplementary Material 1

Supplementary Material 2

## Data Availability

The datasets generated during this study are available from the corresponding author on reasonable request.
