# Supplementary material for: Microvesicles-delivering Smad7 have advantages over microvesicles in suppressing fibroblast differentiation in a model of Peyronie’s disease
Source: BMC Biotechnol. 2024 Jun 7;24:40. doi: 10.1186/s12896-024-00866-1 (PMC11162046; doi:10.1186/s12896-024-00866-1)
Supplement: Supplementary file 1 — Supplementary Material 1 [file 12896_2024_866_MOESM1_ESM.pdf]

**Supplemental figure 1C**

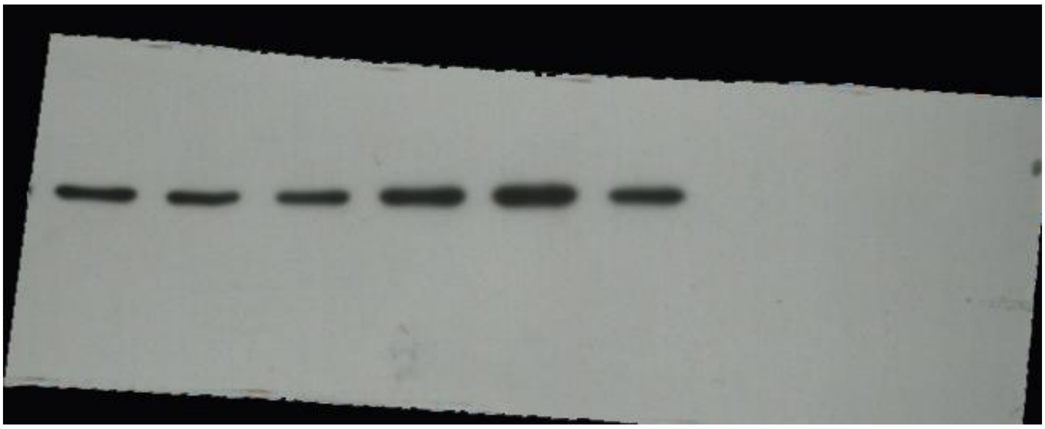

CD63

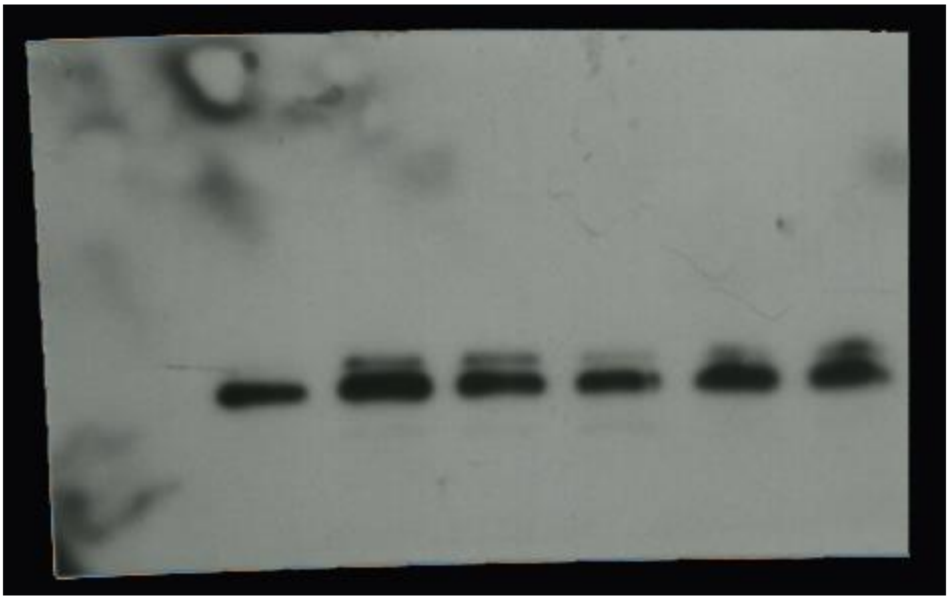

CD81

**Supplemental figure 2B**

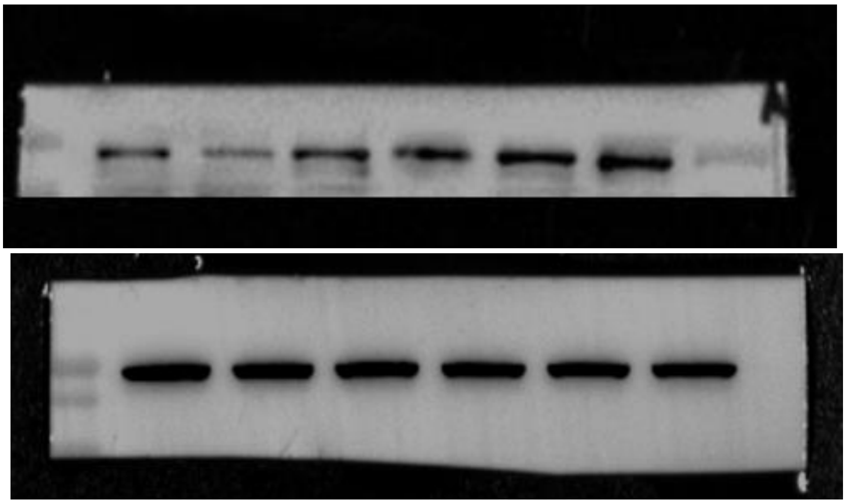

Smad7

β-actin

**Figure 3B**

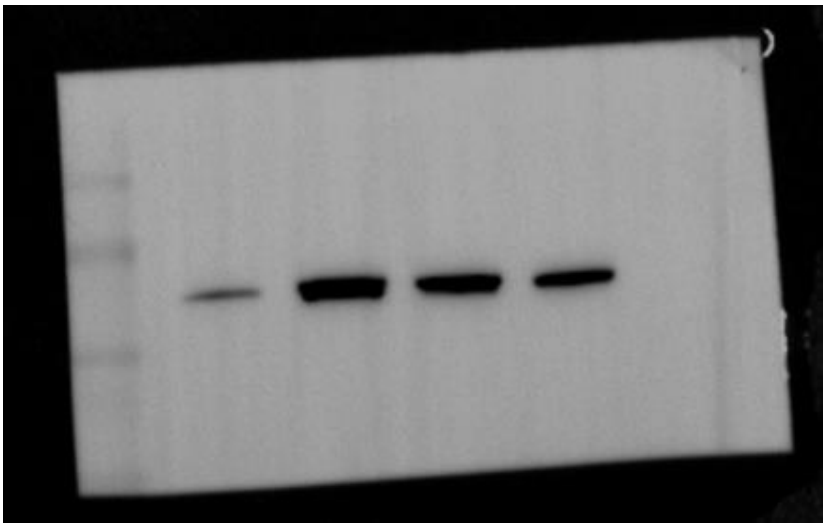

α-SMA

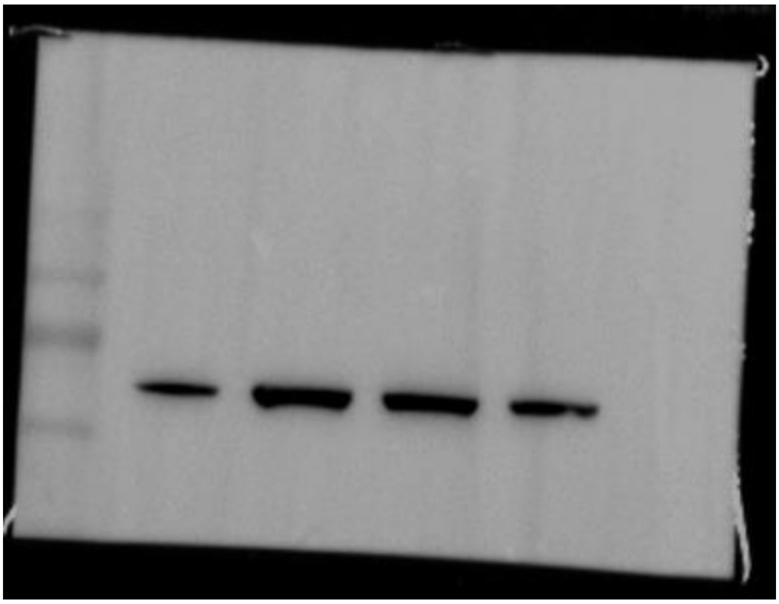

Collagen III

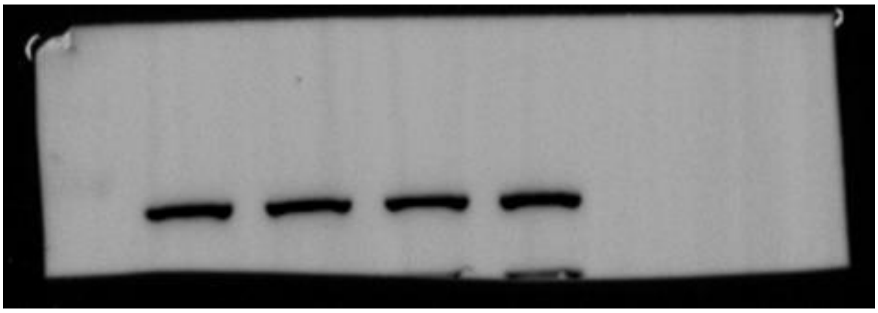

β-actin
