# Supplementary material for: Microvesicles-delivering Smad7 have advantages over microvesicles in suppressing fibroblast differentiation in a model of Peyronie’s disease
Source: BMC Biotechnol. 2024 Jun 7;24:40. doi: 10.1186/s12896-024-00866-1 (PMC11162046; doi:10.1186/s12896-024-00866-1)

Supplemental figure 1 Characterization of microvesicles (MVs) derived from rat bone marrow mesenchymal stem cells (BMSCs). (A) Morphology of MVs was observed under transmission electron microscopy (TEM). (B) Diameter distribution of MVs was analyzed by a qNano particle analyzer. Intensity (%) = the distribution percent of MVs diameter. (C) The levels of specific MVs markers, CD9 and CD63, were measured by western blotting. Transfected BMSCs-derived MVs: BMSCs were transfected with PEI25k/pCMV5-Smad7 vector, and MVs were isolated the transfected cells.

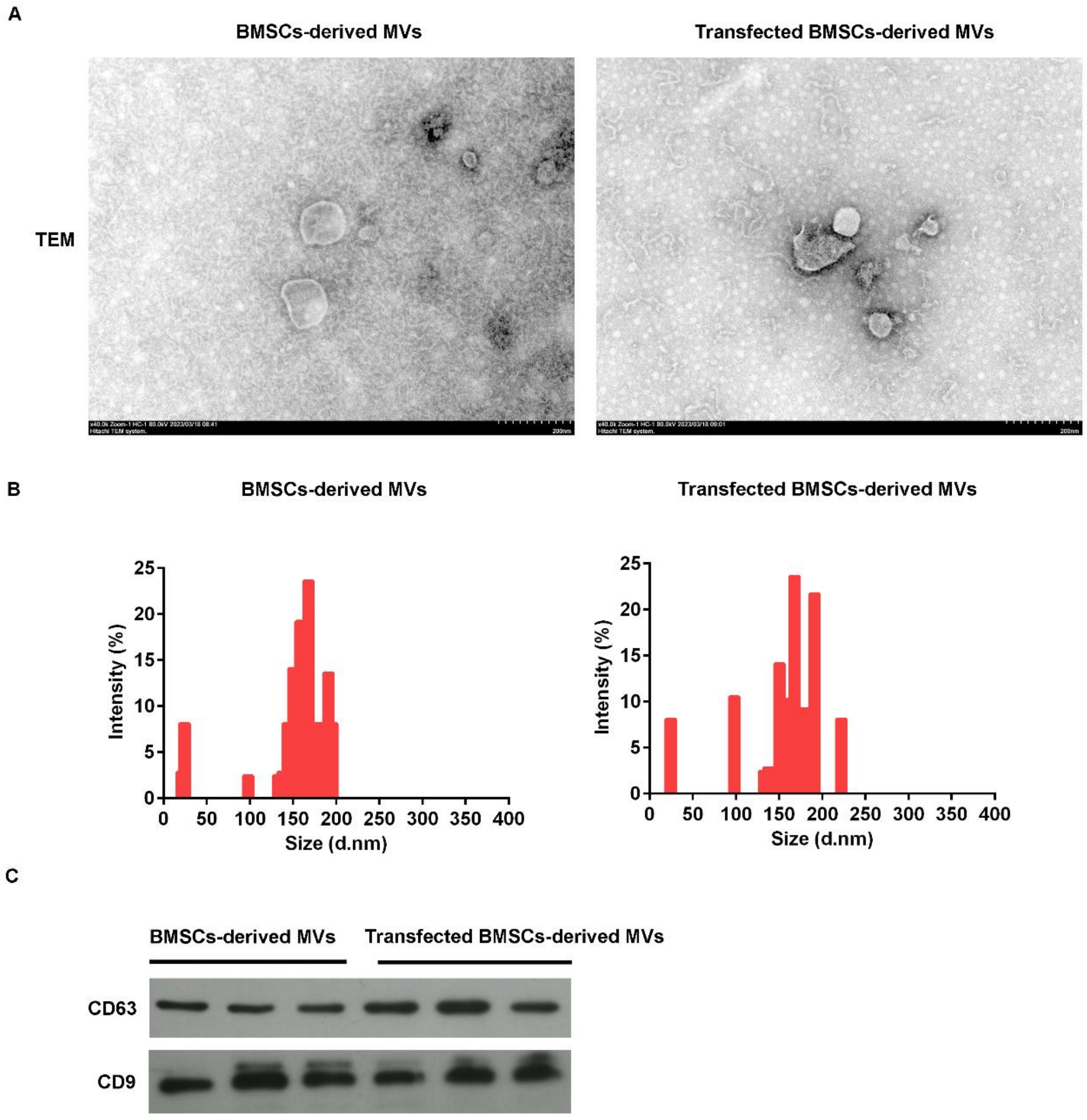

Supplemental figure 2 Delivery of Smad7 protein in MVs. (A) Levels of Smad7 protein were measured by Western blotting. (B) Levels of Smad7 were normalized by β-actin. $^{**}p<0.01$.

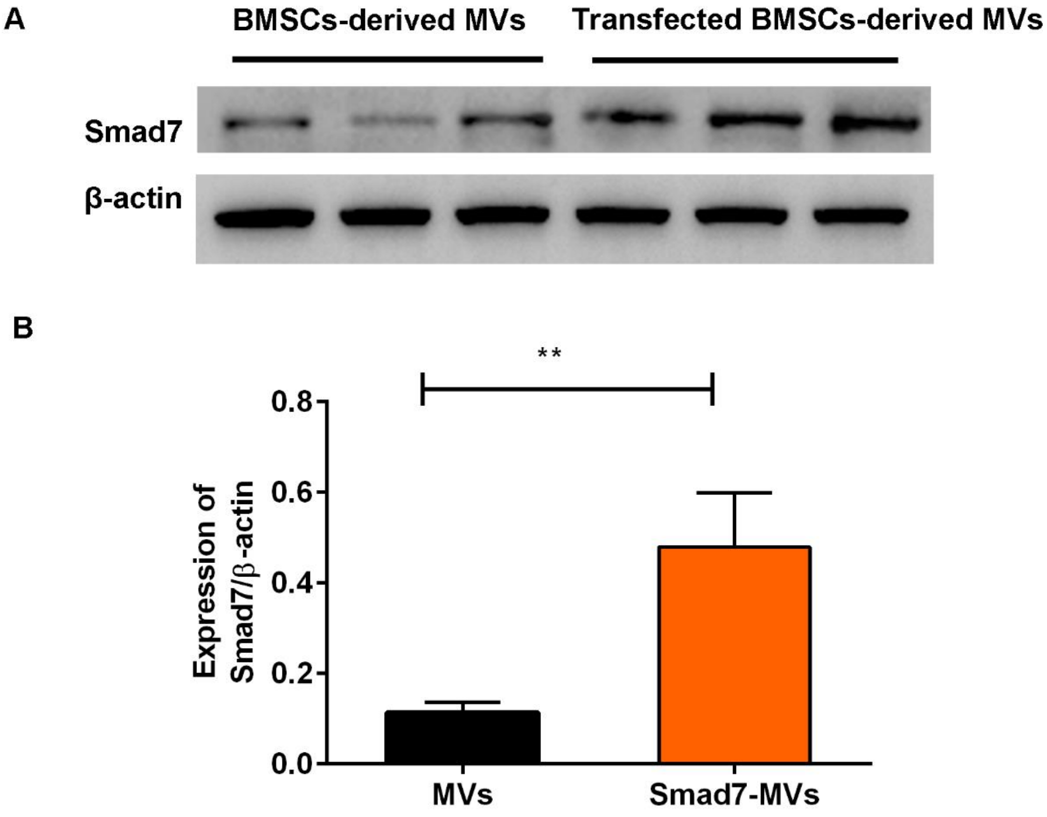

Supplemental figure 3 Biocompatibility of Smad7-Exo in RAW264.7 cells. Cells were respectively cocultured with MVs for 2 h, 12 h and 24 h. Scale = 50 μm. Smad7-MVs: Delivery of Smad7 in MVs.

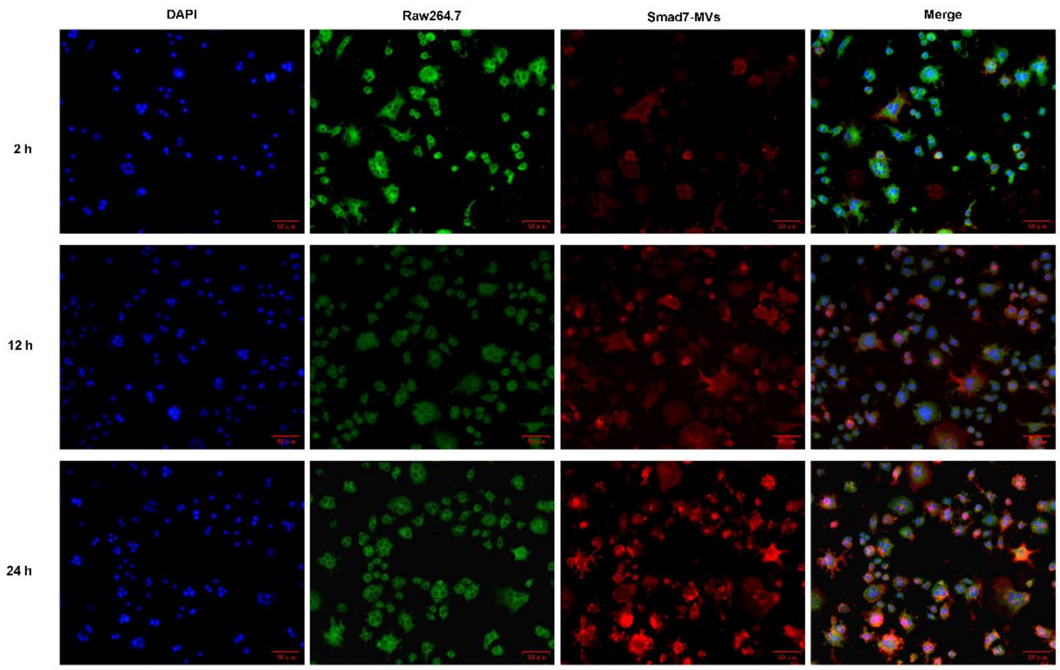

Supplemental figure 4 Biocompatibility of Smad7-Exo in fibroblasts. Cells were respectively cocultured with Smad7- MVs for 2 h, 12 h and 24 h. Scale = 50 μm. Smad7-MVs: Delivery of Smad7 in MVs.

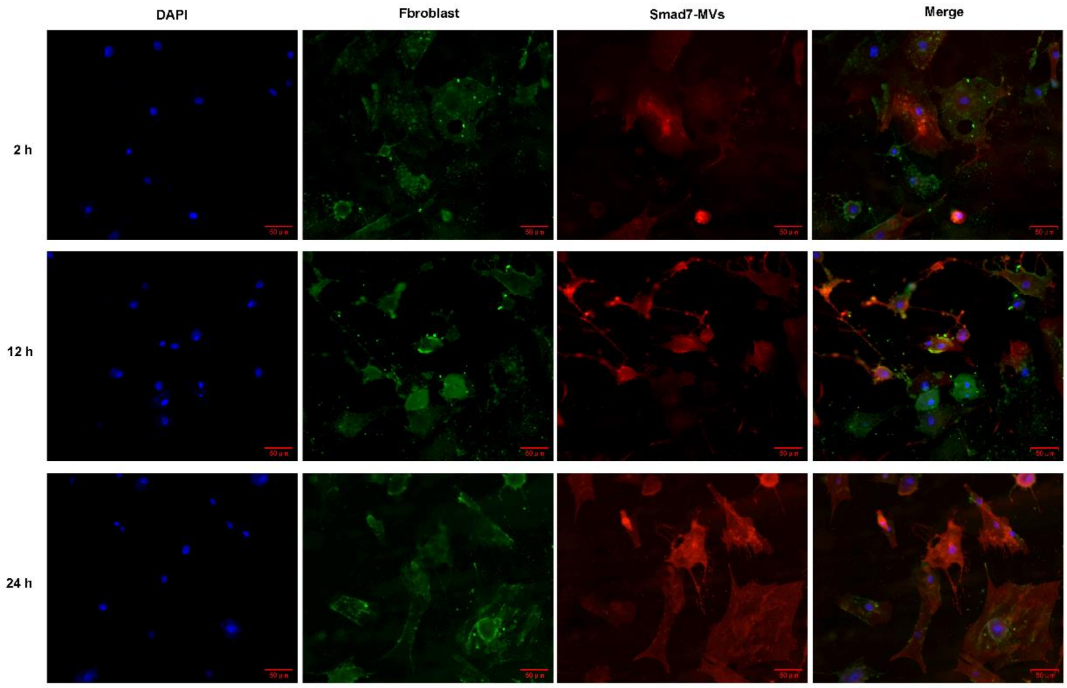

Supplement: Supplementary file 2 — Supplementary Material 2 [file 12896_2024_866_MOESM2_ESM.pdf]
